# An internet-based adolescent depression preventive intervention: study protocol for a randomized control trial

**DOI:** 10.1186/s13063-015-0705-2

**Published:** 2015-05-01

**Authors:** Tracy G Gladstone, Monika Marko-Holguin, Phyllis Rothberg, Jennifer Nidetz, Anne Diehl, Daniela T DeFrino, Mary Harris, Eumene Ching, Milton Eder, Jason Canel, Carl Bell, William R Beardslee, C Hendricks Brown, Kathleen Griffiths, Benjamin W Van Voorhees

**Affiliations:** 1 grid.268091.40000000419369561Wellesley Centers for Women, Wellesley College, Central Street, Wellesley, MA 20481 USA; 2 grid.38142.3c000000041936754XDepartment of Psychiatry, Boston Children’s Hospital, Harvard University, Longwood Avenue, Boston, MA 02115 USA; 3 grid.185648.60000000121750319Department of Pediatrics, the University of Illinois at Chicago, West Taylor Street, Chicago, IL 60612 USA; 4 grid.465264.7Northwestern University Feinberg School of Medicine, East Chicago Avenue, Chicago, IL 60611 USA; 5 grid.413723.0Harvard Vanguard Medical Associates, Cambridge Street, Cambridge, MA 02138 USA; 6 grid.420352.20000000406260188Access Community Health Network, West Fulton Street, Chicago, IL 60661 USA; 7 North Shore University Health System, Pfingsten Road, Glenview, IL 60026 USA; 8 grid.1001.00000000121807477Centre for Mental Health Research, The Australian National University, Eggleston Road, Canberra, ACT 0200 Australia

**Keywords:** Adolescent depression, Prevention, Internet, Adolescence, Primary care

## Abstract

**Background:**

The high prevalence of major depressive disorder in adolescents and the low rate of successful treatment highlight a pressing need for accessible, affordable adolescent depression prevention programs. The Internet offers opportunities to provide adolescents with high quality, evidence-based programs without burdening or creating new care delivery systems. Internet-based interventions hold promise, but further research is needed to explore the efficacy of these approaches and ways of integrating emerging technologies for behavioral health into the primary care system.

**Methods/Design:**

We developed a primary care Internet-based depression prevention intervention, Competent Adulthood Transition with Cognitive Behavioral Humanistic and Interpersonal Training (CATCH-IT), to evaluate a self-guided, online approach to depression prevention and are conducting a randomized clinical trial comparing CATCH-IT to a general health education Internet intervention. This article documents the research framework and randomized clinical trial design used to evaluate CATCH-IT for adolescents, in order to inform future work in Internet-based adolescent prevention programs. The rationale for this trial is introduced, the current status of the study is reviewed, and potential implications and future directions are discussed.

**Discussion:**

The current protocol represents the only current, systematic approach to connecting at-risk youth with self-directed depression prevention programs in a medical setting. This trial undertakes the complex public health task of identifying at-risk individuals through mass screening of the general primary care population, rather than solely relying on volunteers recruited over the Internet, and the trial design provides measures of both symptomatic and diagnostic clinical outcomes. At the present time, we have enrolled N = 234 adolescents/expected 400 and N = 186 parents/expected 400 in this trial, from N = 6 major health systems. The protocol described here provides a model for a new generation of interventions that blend substantial computer-based instruction with human contact to intervene to prevent mental disorders such as depression. Because of the potential for broad generalizability of this model, the results of this study are important, as they will help develop the guidelines for preventive interventions with youth at-risk for the development of depressive and other mental disorders.

**Trial registration:**

Clinical Trial Registry: NCT01893749 date 6 May 2012.

## Background

Adolescent Major Depressive Disorder (MDD) is a significant public health problem. Twenty percent of all adolescents will experience a depressive episode by age 18, with potential adverse impacts on educational attainment, interpersonal relationships, and behavioral health (including increased risk of substance abuse, future depressive episodes, and suicide) [1-3]. This high prevalence in adolescents is concerning, given that depression frequently manifests as a “life-course” disorder, emerging in mid-adolescence, and recurring every 5 to 7 years in 80% of individuals [4,5]. Given the high prevalence of adolescent MDD, the serious associated functional impairments, and the risk of unhealthy behaviors and life-long illness, it is vital to population health that programs are available to address the problem.

Efficacious psychosocial and pharmacological treatments for depression are currently available for adolescents [6,7]. However, adolescents have very low rates of seeking care (35%), completing referrals for psychotherapy (30%), and receiving high-quality treatment (20%) [8-11]. Furthermore, adolescents who do receive high-quality depression treatment do not always recover. Even under controlled research conditions, only about half of adolescents who receive treatment for depression fully recover, and frequently these recovered individuals eventually relapse [12-14]. Given the low recovery rates associated with adolescent depression treatment, and the adverse educational, interpersonal, and behavioral outcomes still present in adolescents who receive depression treatment [1,3], approaches to prevent the onset of adolescent depression are indicated.

Since the 1980s, a number of prevention programs have been developed to reduce the risk of depressive symptoms, episodes, and disorders [15]. More than 30 programs have been developed specifically targeting the prevention of depression in youth, and recent meta-analyses indicate that such prevention programs are effective, particularly when targeting high-risk adolescents [16]. There is substantial evidence that cognitive behavioral approaches, in particular, may prevent adolescent depression [17-20]. However, many barriers associated with depression treatment (for example, high cost, stigma, transportation, reticence to speak with a stranger, and limited availability of providers) are barriers to prevention programs delivered face-to-face [21-24].

The Internet offers promising opportunities for the dissemination of public health adolescent depression prevention programs to overcome the barriers of face-to-face interventions. Use of the Internet to deliver prevention programs enables any adolescent with a web-connection to potentially receive help anonymously, conveniently, and free of charge. Adolescents report extensive use of the Internet [25], and some willingness to utilize health-related websites [26,27]. Therefore, use of the Internet to deliver adolescent depression programs seems a practical and acceptable manner to provide prevention services to adolescents. For adults, Internet-based interventions have been demonstrated to produce clinically meaningful preventive effects, equivalent to face-to-face interventions [28-32], and preliminary research indicates that this is true for adolescent-targeted Internet prevention programs, as well [33]. Despite the many benefits of providing depression prevention programs online, not all Internet-based programs for youth are equally effective [34]. Although adolescents report extensive use of the Internet [25], there are mixed reports on adolescent utilization of health-related Internet websites [25,27], and adolescent participation in Internet interventions has been inconsistent [27,35]. Theory and research indicate that Internet-based programs are most effective when engagement strategies (for example, stimulating material and providing reminders to use the program), both within the intervention website and in real life, are incorporated into the intervention [36-39].

Instructional Design Theory [36] teaches the importance of maintaining learner attention, informing the learner of objectives, and providing opportunities for stimulation, guidance, and feedback for maximizing learning and behavior change. Effective Internet-based depression prevention programs incorporate these principles in their design, and offer additional strategies to promote opportunities for identification and relevance, to engage adolescents in the thought and behavior change process [40].

Motivation and external engagement also are important for Internet-based prevention programs. The literature indicates that adolescents need to be reminded and encouraged to visit intervention websites to sustain use [41,42]. Many effective Internet-based programs for adults use some form of reminder or professional guidance to encourage engagement, and similar protocols also have been effective in adolescent Internet prevention programs [33,37-39]. Recent studies also indicate that motivational interviews with participants linking personal goals to intervention use are more effective at maintaining site use and achieving preventive effects than just brief advice and reminders alone [33].

Cognizant of this research on Internet-based prevention, we developed CATCH-IT (Competent Adulthood Transition with Cognitive Behavioral Humanistic and Interpersonal Training) [43-49] a public health, primary care/Internet-based depression prevention program for adolescents. CATCH-IT teaches resiliency skills to at-risk adolescents through teen-friendly, interactive web-based modules, and incorporates both motivational support by primary care professionals and an Internet-based parental behavioral change course. CATCH-IT targets multiple etiological elements by teaching skills from empirically supported, face-to-face interventions (including Cognitive Behavioral Therapy (CBT), Interpersonal Psychotherapy (IPT), and Behavioral Activation (BA); [33,45,46]), and employs a multi-channel learning process with culturally relevant lessons, stories, and graphics to increase personal relevance and multi-modal learning opportunities. Should this intervention prove efficacious, the public would have the first effective, low-cost, and acceptable depression prevention intervention offered through primary care for young people that would be free to users and available universally, without further burdening the mental health care delivery system or creating any new care delivery infrastructure.

The purpose of this article is to document the study design and research framework used to evaluate CATCH-IT, in order to inform future work in this area. The aims of the CATCH-IT study are to determine (a) whether or not CATCH-IT prevents or delays major depressive episodes, as well as non-affective disorder episodes, compared to a Health Education (HE) control; (b) whether or not CATCH-IT participation is associated with more rapid favorable changes in depressive symptoms and/or vulnerability/protective factors, compared with a Health Education control; (c) whether or not CATCH-IT participation is associated with lower perceived educational impairment, greater quality of life, greater health-related quality of life, and lower incidence of symptoms of other mental disorders (for example, anxiety disorder and substance use disorder), compared with a Health Education control; and (d) for whom and how the CATCH-IT program works in this population. In this paper, we present findings from the multiphase trials of this intervention, and discuss both potential implications of this work, as well as future directions.

## Methods/Design

### Study design

This study is a 5-year, two-site randomized clinical trial to test the efficacy the CATCH-IT preventive intervention against the Health Education (HE) control program in preventing the onset of depressive episodes in an intermediate to high risk, geographically representative sample of adolescents aged 13 to 18.

We identify high risk adolescents based on elevated depressed mood scores (cut-off set to maximize sensitivity and specificity for future episodes; [14,50,51]). Enrolled adolescents are randomized into either the CATCH-IT or the HE group, and are assessed at baseline and at 2, 6, 12, 18 and 24 months post-intake on measures of depressive symptoms, depressive diagnoses, other mental disorders, and on measures of role impairment in education, quality of life, attainment of educational milestones, and family functioning in order to examine predictors of intervention response. Ethical bodies that approved this study are discussed in the Acknowledgements section.

### Primary care site recruitment and implementation process

The primary care aspects of the study (including recruitment, provider motivational interview, and referrals to treatment, when clinically indicated) are centered in pediatric clinics in urban and suburban areas in both Chicago, IL and Boston, MA. Practices are recruited either by study staff or by health care providers at existing study sites. Healthcare providers (physicians and nurse practitioners) and office nursing staff (for example, registered nurses and medical assistants) are informed about the study and provide written consent to participate in specific roles described below. Each participating healthcare provider also completes pre- and postquestionnaires regarding the intervention, status of the clinic, and their experiences in participation. At each site, a research coordinator or study champion is identified to communicate with study staff about room scheduling, weekly recruitment times, positive and negative screens, and other study details. Providers and other medical professionals are instructed in the screening protocol. Office nursing staff conduct the screenings of the adolescents with the two question screening tool. Health care providers are trained in Motivational Interviewing either with a one hour program using a lecture/discussion format and example video tapes, a three-session training, or through more informal interactions with the study staff, using detailed scripts. Providers receive semi-structured feedback on their motivational interview technique after completing 1 to 2 interviews with teen participants at their clinic. Each motivational interview takes approximately 10 minutes to complete. Three interviews are completed over the course of the year with each participant, with each provider interviewing one to eight patients. Study-employed mental health professionals, who conduct three motivational phone calls with the participants during the intervention, are also trained in Motivational Interviewing. Throughout the study, study staff members are in frequent contact with the primary care clinics, discussing logistical details, reporting when screened adolescents require referral to treatment, and providing feedback about the clinic’s participation.

### Inclusion criteria

Adolescents (ages 13 through 18) experiencing elevated levels of depressive symptoms on the Center for Epidemiologic Studies Depression (CES-D; [50]) scale are eligible for the study (eligible scores are between 8 to 17, inclusive, on the ten-item shortened scale; those with scores of 18 to 20 are considered with permission of the principal investigator). Adolescents also are included if they have a past history of depression or dysthymia.

### Exclusion criteria

Adolescents must not have any of the following: (a) a current DSM-IV diagnosis (Kiddie Schedule of Affective Disorders [K-SADS]; [52]) of Major Depressive Disorder, current therapy for depression, or be taking antidepressants (for example, SSRIs, TCAs, MAOIs, bupropion, nefazodone, mirtazapine, or venlafaxine); (b) a history of being treated with Dialectical Behavioral Therapy or eight or more sessions of Cognitive Behavioral Therapy; (c) a current CES-D score of outside the range of 8 to 20 on the short form or 16 to 35 on the long form [50]; (d) a DSM-IV diagnosis of schizophrenia (current or past) or bipolar disorder; (e) a current serious medical illness that causes significant disability or dysfunction; (f) a significant reading impairment (a minimum sixth-grade reading level based on parental report), mental retardation, or developmental disabilities; (g) a serious imminent suicidal risk (as determined by endorsement of current suicidality on the CES-D or in the K-SADS interview) or other conditions that may require immediate psychiatric hospitalization; (h) psychotic features or disorders, or currently be receiving psychotropic medication; or (i) extreme, current drug/alcohol abuse (greater than or equal to 2 on the CRAFFT Screening Test; [53]).

### Participant recruitment and enrollment

Participants are recruited through participating primary care clinics; all adolescent patients are provided with a brief description of the study while visiting the clinic, along with a study brochure. Recruitment letters sent to patient homes are also used as a form of recruitment along with posted study flyers. Participants are identified using a public health model of screening, whereby all adolescent patients are screened for risk (subthreshold depressed mood) while visiting their primary care clinics, thus encompassing diverse populations in urban and suburban areas in Chicago and Boston. We use a simple two-question screener (depressed mood/irritability, and/or anhedonia for at least 2 weeks), based on the Patient Health Questionnaire-Adolescent [33,54]. If an adolescent responds positively to a screener item, initial parental consent and adolescent assent are obtained to conduct an eligibility assessment by phone using the CES-D and a checklist of survey exclusion criteria. Some clinics, rather than presenting teens with a two-question screener in the office, have opted to send letters about the study to all teens in their practice. Upon receiving the letter, teens have called for further study information. After parents have given consent, these teens also have participated in an eligibility assessment by phone. After establishment of eligibility criteria, the parent and adolescent are scheduled for an enrollment assessment interview (which includes informed consent, the K-SADS interview, and completion of other psychometric scales) at their primary care office to confirm final eligibility. Enrollment and randomization of subjects is then completed [55]. Primary care providers are supported with materials and training to respond to adolescents identified with major depression during screening or during study participation.

### Sample size

Our intent is to recruit and randomize 400 participants (200 per site). We anticipate being able to retain 80% of these youth throughout the two-years of follow-up. In Van Voorhees’s pilot study of the CATCH-IT intervention, of the 84 randomized participants, only 7 did not take part in any follow-up (91% retention) [5]. We implemented standard measures to minimize attrition: all adolescents who express challenges related to study participation are contacted by research staff; attempts are made to resolve their concerns so they can continue to participate; and standard methods are used to optimize retention (for example, birthday cards and regular updates on contacts). In keeping with an intent-to-treat design, all randomized cases who provide consent are followed and included in data analyses.

### Randomization

Participants are assigned randomly to CATCH-IT or HE. Randomization was determined by a computer generated sequence and was blocked by practice and time of entry, and stratified by level of risk severity (based on CES-D score, prior episodes of either major depression or dysthymia), age (13 to 14 or 15 to 18) site (Chicago or Boston) [56,57].

### The CATCH-IT intervention

The CATCH-IT intervention has an Internet component (with separate adolescent (14 modules) and parent (4 modules) programs) and a motivational component (three primary care physician motivational interviews at time 0, 2 months and 12 months, and one to three study-staff coaching phone calls either at 1 month (Chicago) or at 2 and 4 weeks and 18 months (Boston)). In addition to the motivational interviews and coaching phone calls, three “check-in” calls during weeks 1 to 3 are offered to participants; these calls are intended to ensure participants have access to the Internet and take approximately five minutes to complete.

### CATCH-IT: The adolescent internet component

The adolescent Internet component comprises 14 modules that teach skills from BA, CBT, IPT, a community resiliency concept model [19,46,48,58-60], and an optional anxiety module. The intervention is intended to reduce cognitions (for example, dysfunctional thoughts, impaired problems solving, or pessimistic expectations), behaviors (for example, procrastination, passivity, and avoidance), and interpersonal interactions (for example, indirect communications) that are associated with increased risk of depression, and to strengthen behaviors (for example, behavioral scheduling of pleasurable activities), thoughts (for example, optimistic appraisals, counter thoughts, and effective problem solving), and interpersonal relations (for example, effective social problem solving, building and engaging social support) thought to be protective against depression [33].

#### Development and modification

The development of CATCH-IT was heavily informed by research and theory (for example, [47]). Each module was designed to address the objectives of Instructional Design Theory [36], such as gaining learner attention, strengthening recall, and increasing retention; this was accomplished by creating identically structured modules that each include a lesson overview, review of previous content, core concept explanation, adolescent stories (including scripted video “diaries”) to illustrate the lessons, skill building and self-efficacy exercises, a summary, feedback on the experience, and an Internet-based reward (gift card).

To address the need to engage adolescents with varying levels of motivation, we grounded the intervention in key principles of effective community-based preventive interventions: sufficient dose, training, positive relationships, and sociocultural relevance [61]. The adolescent stories, designed to stimulate recall, understanding, and retention, were written in first person or intimate third person style so as to connect directly with the experience of the reader. These stories were included based on both the principles of Nation’s (2003) socio-cultural relevance and of Vicarious Learning Theory [36]. Instructional Design Model, the Trans-Theoretical Model of Change, and the Theory of Planned Behavior [47] informed the modification of the original adolescent Internet component. Through the modification process, text was shortened and translated into “teen-friendly” language, and videos were included. These modifications were made using previously published methods [47,55,62].

#### Lessons and structure

The CATCH-IT online intervention comprises fourteen core modules, as well as an optional anxiety module, and online tools to track mood (a feedback mechanism), manage intense feelings, and solve problems that are interfering with the completion of the program. The fourteen core modules of CATCH-IT are grouped into six sections: Introduction, How Do You Act, How Do You Think, How Do You Socialize, How Resilient Are You, and Wrap Up. Each section contains one to four modules. Each module is approximately 20 slides long, and designed to be completed in approximately 15 to 20 minutes.

##### Section one: Introduction (Module 1)

The first CATCH-IT section comprises one module, titled “How Can This Help?” This module introduces the concept of CATCH-IT and prompts adolescent participants to think about their goals and how using CATCH-IT can help them achieve these objectives (by improving mood, solving relationship problems, building social support, or increasing enjoyable activity in one’s routine). Information is provided about the structure of each following module, as well as about special CATCH-IT tools to track mood, manage intense feelings, and solve problems interfering with the completion of the program. The module concludes by previewing the concepts of Behavioral Activation (BA), Cognitive Behavioral Therapy (CBT), and Interpersonal Therapy (IPT).

##### Section two: How do you act (Modules 2 to 4)

The second CATCH-IT section, “How Do You Act,” comprises three modules: “Going Outside In,” “Habits, Acting & TRAPS,” and “Choosing To Get Back On Track,” which instruct participants in principles of BA.

“Going Outside In” introduces the BA approach. Adolescent participants are instructed that negative mood can result from specific situations in which one feels a lack of achievement or pleasure, and that adjusting situations to increase feelings of mastery and pleasure can correspondingly improve mood. Participants are encouraged to track throughout the week how much mastery and pleasure their daily activities evoke, and then plan new activities in their routines that will increase their sense of mastery or pleasure.

“Habits, Acting & TRAPS” discusses how maladaptive habits of avoidance and procrastination can prolong feelings of sadness and stress, which can create a cycle of further avoidance and resulting poor mood. The module introduces the TRAP (Trigger, Response, Avoidance Pattern) concept, which teaches that specific situations, or triggers, evoke negative feelings (response), which compel individuals to avoid the trigger (avoidance pattern). This module provides examples of avoidance patterns (such as habitual napping, skipping activities, and procrastinating) and encourages participants to identify what situations in their lives trigger them, how the triggers make them feel, and what avoidance patterns result.

“Choosing To Get Back On Track” encourages participants to choose alternative coping behaviors, such as breaking a task into small parts or rewarding one’s self for addressing a triggering situation, instead of avoidance behaviors. The module teaches participants how to choose and integrate alternative coping behaviors into their routines. The module also addresses how to use BA when participants experience low energy, lack of motivation, or more significant personal problems.

##### Section three: How do you think (Modules 5 to 8)

The third CATCH-IT section, “How Do You Think?” comprises four modules focused on Cognitive Behavioral Therapy (CBT) skills: “Freedom From Negative Thoughts,” “Changing Your Thoughts And Feelings,” “How To Figure Out What Bothers You,” and “Problem Solving In Stressful Situations.”

“Freedom From Negative Thoughts” provides an introduction to the theory behind CBT, explaining in adolescent-friendly language how negative thoughts can create low mood and result in maladaptive behaviors.

“Changing Your Thoughts And Feelings” begins with instruction in the ABC (Activating event, Belief, Consequence) method, which involves identifying the activating event that triggers poor mood, the negative belief associated with the trigger, and the emotional consequence of the negative belief. Adolescent participants are encouraged to challenge negative beliefs about an activating event with counter-thoughts that are more positive. Participants are encouraged to choose their most negative belief each day and write down a counter-thought to reframe the activating event in a neutral or positive manner. Participants are reminded to reward themselves when they change their thinking.

“How To Figure Out What Bothers You” begins with a motivational component encouraging participants to review how far they have come in the program and to reward themselves for their progress. Next, the module instructs participants in the CAB (Consequence, Activating event, Belief) method, in which adolescents identify their negative mood, the event that preceded this mood, and the negative belief about the event that led to the negative feelings. Adolescents are encouraged to replace their belief with a counter-thought that improves their feelings about the situation. Instruction is provided on how underlying beliefs can cause one to have negative beliefs after an activating event, and how these beliefs can be identified and addressed. Examples of counter-thoughts are provided for common negative underlying beliefs.

“Problem Solving In Stressful Situations” provides tips on reducing low frustration tolerance, avoiding negative people, stopping negative thoughts, changing difficult situations, and changing one’s emotional response to stressful situations. Participants are encouraged to diagram stressful situations in their lives, predict their reactions to them, and develop a coping plan.

##### Section four: How do you socialize (Modules 9 to 12)

The fourth CATCH-IT section, “How Do You Socialize,” comprises four modules that teach Interpersonal Therapy (IPT) skills.

“Relationship Skill Training” discusses the importance of relationships and the role of IPT in improving social skills and decreasing negative feelings associated with social problems. Participants are encouraged to map out patterns of strengths and areas for improvement in their relationships with specific individuals.

“How Do I Communicate” discusses different communication styles, provides a quiz to identify the user’s style, and provides insight on how others may react to each approach. The module emphasizes the benefits of responding to frustrating situations in a calm, clear manner, and of listening to the perspective of others to resolve conflict.

“Relationship Conflicts” discusses how differences in expectations and poor communication can cause conflict. Participants are encouraged to keep a relationship diary to observe patterns in their interactions. Step-by-step instruction is provided on identifying conflicts in relationships and addressing them constructively.

“Dealing With Real Life Situations” describes how relationships can change due to life changes, and how life changes create an opportunity to take on new roles. Instruction is provided on defining old and new social roles, evaluating the skills needed for the new role, and finding social support for the new role.

##### Section five: How resilient are you (Module 13)

The fifth CATCH-IT group, “How Resilient Are You,” comprises a single module, “Building a Resilient Life,” which teaches skills from a community resiliency concept model.

“Building a Resilient Life” teaches four resiliency skills: first reactions, ways of living, organized coping, and surviving bad situations. First reaction skills taught include building a sense of humor, refocusing energy on constructive activities, taking a break, and prioritizing problems. Ways of living refers to setting goals, problem-solving challenges, and fulfilling needs for companionship and fun. The organized coping section instructs that optimism and hope are important tools for solving problems. Surviving bad situations describes how successful survivors of tragedy do not blame themselves for the acts of others and can grow stronger from traumatic situations by seeking help and realizing they can take control over their own lives. Creativity, resourcefulness, being involved in meaningful activities, finding one’s place in society, and being a part of a community are highlighted as characteristics of resilient people.

##### Section six: The wrap-up (Module 14)

The final CATCH-IT section has one module, “What To Do If You Can’t Shake The Blues.” This module describes clinical depression, how to stop the process of developing depression, and information about the identification and treatment of major depressive disorder.

##### Optional anxiety module

An optional anxiety module is provided, which teaches relaxation techniques of muscle relaxation, visualization, and deep breathing.

### CATCH-IT: The parent internet component

The parent Internet component of the intervention is based on an adaptation of Beardslee and Gladstone’s clinician-facilitated and lecture intervention approaches from the Preventive Intervention Project [43]. This intervention helps parents develop the awareness and skills needed to help build resiliency in their children. The intervention also seeks to reduce known risk factors for adolescent depression. To address the key mediating role of parental depressed mood, a personalized component is available to parents with depressed mood. A paper and pen version of the parent component was originally piloted during the phase 2 CATCH-IT clinical trial, but parents strongly requested their own Internet site. A paper version of the current parent program is available in Spanish.

#### Lessons and structure

The parent Internet component of CATCH-IT comprises four modules, with an additional fifth module that is optional. Each module is approximately eight slides in length.

Module One describes the goals of CATCH-IT, the concepts of BA, CBT, and IPT, and how to encourage a child to finish the CATCH-IT program. Module Two describes depression symptoms, causes, and treatment methods. Module Three discusses how to recognize depression in children and adolescents, invites parents to reflect on how their child is currently functioning, and provides instruction on actions to take if one’s child might be depressed. Module Four describes characteristics of resilience in teens and strategies for promoting resilience in one’s adolescent and family. The optional fifth module discusses what to do if a parent believes they are depressed, how parental depression is associated with depression risk in offspring, and how to promote health and well-being in a teen while struggling as a parent with depression. This module specifically provides family-based prevention strategies adapted from Beardslee and Gladstone’s Preventive Intervention Project [43].

### CATCH-IT: The motivational component

The CATCH-IT intervention uses motivational interviewing, goal-setting, and telephone coaching to enhance the change process and to advance the participants through the stages of change to reduce vulnerability and increase protective factors [63-66]. In the adolescent motivational interview (10 to 15 minutes duration), the primary care provider seeks to help the adolescent weigh the balance of positives and negatives of undertaking this depression preventive intervention. The coaching phone calls are conducted by study staff and use the same motivational interview approach, but last 5 minutes or less in duration and are solely designed to encourage completion of the intervention and behavior change. All adolescents are asked to complete a motivational interview questionnaire before the interview to enhance level of participation and fidelity to the interview.

### Control group: health education

Health Education (HE) is an attentional control. This Internet site comprises 14 modules, 13 of which provide instruction on nutrition, safety, and other teen health and wellness topics. The 14th module discusses mood and seeking mental health treatment, and also addresses stigma and negative attitudes toward the treatment of mental disorders, as these have previously been identified as barriers to seeking and adhering to treatment [47,67]. Module 14 does not provide instruction in Behavioral Activation, Cognitive Behavioral Therapy, or Interpersonal Therapy. The HE Internet site does not include interactive elements such as videos, question prompts, or feedback on mood or progress.

The HE components are similar to those employed in previous primary care-based quality improvement/Chronic Care Model Interventions, incorporating patient psychoeducation, active monitoring and referral, with routine contact with a primary care provider. The HE group receives the same assessments as the CATCH-IT group (including notification and referral to mental health services) and routine contact with their primary care provider (PCP; estimated two to three visits per year), and also incorporates a parent Internet program that provides similar psychoeductional content about general adolescent health. Psychoeducation Internet site use is monitored to compare with use of the CATCH-IT intervention as an attentional control. Self-harm risk is managed based on a standard protocol.

### Instruments

#### Screening measures

##### Two-question screener

The two-question screener was developed by study staff to screen for possible depressive symptoms. The two-item self-report tool, based on the Patient Health Questionnaire-Adolescent [33,54] asks adolescents to endorse whether or not they have been experiencing depressed mood/irritability or anhedonia for at least the past 2 weeks. The questions include “Have you had any of the following problems during the last 2 weeks? 1) Little interest or pleasure in doing things? 2) Feeling down, depressed, irritable or hopeless?” There are three possible responses to each item: 1) Yes: Nearly every day in the past 2 weeks; 2) Yes: a few days in the past 2 weeks; Or 3) No. Any endorsement qualifies the adolescent as a positive screen. Providers at participating practices invite all adolescents of the appropriate age to complete the screener.

##### Phone screen interview

The phone screen is a 10- to 15-minute scripted interview developed by study staff and conducted over the phone with the adolescent (those who screened positively on the two-question screener or who contacted the study team in response to a letter about the study from their pediatrician). After parental permission and assent (or consent for those age 18) are provided, adolescents are then asked about the presence of exclusion criteria. This includes a checklist of disqualifying characteristics (a sample item is “Are you currently in counseling for depression?”) and a subsequent depression symptom checklist, to screen for adolescents who meet criteria for current major depressive disorder (five or more symptoms; A sample item is “Has there been a 2-week period of time in the past 2 months when you have been feeling down, depressed, or sad much of the time?”). Adolescents who do not endorse these preliminary exclusion criteria are then administered the short, ten-item version of the CES-D (see description below) to assess current depressive symptoms; scores above 20 are excluded from the study and given referral to treatment, and scores of 8 to 20 (18 to 20 on a case-by case basis) are eligible for the in-person K-SADS interview (where final eligibility for the study is determined), and scores below 8 are provided with a final three question screen for past depressive episode (a sample item is “Has there ever been a time in your who life when for at least 2 weeks, you felt down, depressed, or sad for much of the time?”). Those who scored below 8 on the short CES-D but endorse a positive history of depressive episode are eligible for an in-person K-SADS interview, and those who do not are excluded.

#### Psychopathology and treatment

##### Kiddie Schedule for Affective Disorders Scale

The Kiddie Schedule for Affective Disorders Scale (K-SADS [52]) is a reliable and valid semistructured clinical interview used to assess current and lifetime psychiatric diagnoses in participants under age 18 [52]. The psychometric properties demonstrate test-retest reliability estimates of diagnoses in the excellent to good range [52]. We use the entire section covering depression and the brief screening questions for bipolar disorder, psychosis, and substance abuse (the section for anxiety is not used). The child is interviewed and then the parent is interviewed about the child, thereby yielding two sources of information about the child’s functioning. The K-SADS measures the occurrence, degree of severity, and the extent of impairment, using the Depression Rating Scale (DRS), which yields a depression score from 1 to 6 for current symptoms as well as worst past episode and first full episode. We establish if a participant meets criteria for major depressive episode (DSR 4 or above) and major depression disorder (DSR 5 or above).

##### Kiddie Longitudinal Interval Follow-up Evaluation

The Kiddie Longitudinal Interval Follow-up Evaluation (K-LIFE [68]) is an adaptation of the K-SADS that provides information about psychiatric diagnoses during the interval since the previous assessment. It is used at all follow-up assessments. Children and parents are questioned about all symptoms present at the previous assessment and about any new symptoms that may have developed since they were last interviewed, and both reports are considered in determining K-LIFE ratings. K-LIFE is reported to have high reliability [68]. Depression Severity Ratings (DSRs) are obtained for each week of the follow-up interval.

##### Child and Adolescent Service Assessment

The Child and Adolescent Service Assessment (CASA [69]) is used to record psychiatric service utilization and access to mental health services within the past 3 months (or, in the case of follow-up interviews, the interval since the previous assessment). The CASA consists of two parts: the Screen and the Detailed Services Form. The Screen covers a broad range of mental health services, including informal ones such as seeking support from a school guidance counselor or talking to a youth minister. The Detailed Service Form is used to provide detailed information for some of the service endorsed on the screen, and includes items such as the number and length of visits, focus of treatment, and if medication was prescribed. The CASA is administered to both the adolescent and the parent about the adolescent at baseline and at all follow-up interviews. The CASA was selected for this study with two purposes in mind: to determine if the adolescents we identified as having a psychiatric illness are actually receiving treatment, and from a public health standpoint, to ascertain the cost to society of the illnesses CATCH-IT aims to prevent.

#### Vulnerability- symptoms

##### Center for Epidemiological Studies-Depression Scale

The Center for Epidemiological Studies-Depression Scale (CES-D [50]) is a self-report measure of the frequency of 20 depressive symptoms over the past week, using a four-point scale. This measure is used with both adolescents and parents about themselves. Sample items include “I was bothered by things that usually don’t bother me” and “I could not get going.” The use of self-report scales like the CES-D as depression case-finding or screening instruments has been successfully validated with both adults [70,71] and adolescents [72,73]. The CES-D is short and easy to read, has been successfully administered in several large adolescent school samples [73,74], and has strong psychometrics with youth [75]. In our pilot study, both baseline and follow-up Cronbach alphas were .91 [5,33].

##### The Screen for Child Anxiety Related Emotional Disorders

The Screen for Child Anxiety Related Emotional Disorders (SCARED [76]) is a 41-item scale, with five factors (somatic/panic, general anxiety, separation anxiety, social phobia, and school phobia) corresponding to different diagnostic categories of anxiety on a 3-point scale (0 = *not true/hardly ever true*, 1 = *sometimes true*, 2 *= true/very true*). A sample item is “I worry about the future.” The SCARED scale has good reliability and validity [76]. The adolescent completes this self-report, and the parent also completes it about their adolescent. The SCARED has good reliability (α = 0.74) and validity [76].

##### The Disruptive Behaviors Disorder Scale

The Disruptive Behaviors Disorder Scale (DBD [77]) form is a 45-item self-report measure assessing the level of teens’ behavioral problems, rated on a four-point scale (0 = *not at all* to 3 = *very much*). A sample item is “often loses temper.” This scale is used with both the teen and the parent about the teen. This scale has shown good reliability and validity [77].

#### Vulnerability - cognition, social factors, and adverse events

##### The CRAFFT

The CRAFFT [5] is a six-item measure that reports the frequency of the use of drugs to relax, use of drugs when alone, driving while using drugs/riding in a car with a driver who is using drugs, family or friends concern about drug use, and negative consequences encountered for using drugs (α = 0.68) [53]. The CRAFFT has good discriminative properties for detecting substance-use disorders in adolescents [78]. The CRAFFT is only completed by adolescents.

##### The Adolescent Life Events Questionnaire

The Adolescent Life Events Questionnaire (ALEQ [79]) is a retrospective self-report measure that asks the teen to report on the presence/absence of various life events in the following categories: health/loss, conflict/arguments, changes/moves, school/job, finances, and crime and legal issues. The internal consistency reliability of the ALEQ was reported as 0.94 [79].

#### Relationships

##### Sibling Relationship Questionnaire

The *Sibling Relationship Questionnaire (SRQ* [80]*)* and the corresponding parent SRQ are used to assess the nature of a child's relationship with his or her siblings. This 48-item measure includes 16 scales of three items each. Children are asked to indicate on a five-point scale (1 = *hardly at all* to 5 = *extremely much*) how prevalent certain qualities are in their sibling relationship. Parents provide the same information about the target child and that sibling. The SRQ has been used for children and adolescents aged 8 to 18, and it has been shown to have acceptable internal consistency, all greater than 0.60, as well as test–retest reliability and adequate construct validity [80].

##### Sibling Inventory of Differential Experience

The Sibling Inventory of Differential Experience (SIDE; [81]) is a nine-item questionnaire that asks adolescents to compare their experiences to the experiences of their siblings in three different areas: parental treatment, sibling treatment, and peer characteristics. For this study, we are only using the nine items of the parental treatment section, which assesses parental affection and control. The SIDE shows adequate psychometric properties; the test-retest reliabilities have a mean of 0.84 [81].

##### Child Report of Parental Behavior Inventory

The Child Report of Parental Behavior Inventory (CRPBI; [82]) measure contains 15 items inquiring about the child’s relationship with his/her mother and 15 items about his/her relationship with his/her father. The items are rated *“not like him/her,” “somewhat like him/her,”* or *“a lot like him/her.”* It assesses interpersonal relationships between parents and their children and taps into parenting styles and nurturing behaviors. The CRPBI correlates well with other measures of parenting. Scores suggesting parenting impairments have been linked to both parental and child psychopathology [83]. The CRPBI is given to both adolescents and parents.

##### Conflict Behavior Questionnaire

The Conflict Behavior Questionnaire (CBQ; [84]) contains 20 true/false items that pertain to the degree of negative communication and conflict in families during the past 2 weeks. Items are rated as 0 = *false* or 1 = *true*. Higher scores reflect greater levels of conflict. This measure is completed by parents, who report on their own parenting behaviors, and by adolescents, who report on their parents’ parenting behaviors. Robin and Foster reported a 6- to 8-week retest reliability of .57 and .84 for these scales [85].

##### Physician Relationship Scale

The Physician Relationship Scale [33] contains nine items rating the participant’s relationship with his/her provider in understanding, engagement, helpfulness, comfort, and trust. Only adolescents in the CATCH-IT condition are administered this measure. This self-report measure is rated on a five-point Likert scale (1 = *strongly disagree* to 5 = *strongly agree*). A sample item is, “I trust my physician.” Higher scores reflect a more positive relationship with the provider. In our pilot study, Cronbach alpha was .85 [5,33].

#### Functional status

##### Pediatric Quality of Life and Enjoyment and Satisfaction Questionnaire - parent and child versions

On the Quality of Life and Enjoyment and Satisfaction Questionnaire - parent and child versions (PQ-LES-Q [86]) questionnaire, adolescents and parents (who respond about their adolescent) are asked to rate their/their child’s life experience on 13 items on a six-point Likert scale (1 = *very poor* to 6 = *very good*). A sample item is “In general, I would rate my health as…” In terms of reliability, the Cronbach alpha has been demonstrated to be excellent (alpha = .90; [86]). In terms of validity, this measure demonstrates a significant correlation with the Children’s Depression Rating Scale-Revised [86].

#### Developmental milestones

##### Masten’s Status Questionnaire

We will assess developmental task attainment at 24 months using adolescent response to the 102-item Masten’s Status Questionnaire [87]. Competence is defined with respect to effective functioning in age-salient developmental tasks. Part of our examination of role functioning will focus on four of the six developmental tasks as delineated by the Masten’s Status Questionnaire: academic achievement (*alpha = 0.9*), work (*alpha = 0.74*), social competence with peers (*alpha = 0.86*), and romantic relationships (*alpha = 0.77;* [87]). The other two developmental tasks, parenting and conduct, will not be assessed, as relatively few of our participants will be parents and because we access externalizing symptoms in our measures of mental health outcomes. The Masten’s Status Questionnaire has excellent predictive validity [88].

#### Motivation

##### Theory of planned behavior scale

This 19-item instrument, originally developed for prostate cancer, was adapted to primary care-based depression prevention (α = 0.76) [89]. The adolescent participants indicate a level of agreement based on a five-point Likert scale (1 *= strongly disagree* to 5 *= strongly agree*) with items such as “depression intervention makes sense to me.”

##### Trans-theoretical model scale

We measure adolescent motivation to change risk-factor behaviors before, during, and after the intervention. We adapted the standard approach for measuring motivation as described by Miller and Rollnick [90] to evaluate importance (“rate the importance of preventing a depressive episode”), self-efficacy (“rate your ability to learn coping skills to reduce your risk of depression”) and readiness (“rate your readiness to learn coping skills”) on a 10-point Likert scale (1 = *not important* to 10 = *very important*). The Cronbach’s alpha was reported as 0.76.

#### Training

##### Usability and clarity

Adolescent participants respond to questions about ease of use (that is, “this module was easy to use”), ease of understanding (that is, “this module was easy to understand”), and ease of reading (that is, “this module was easy to read”) of the online intervention. Items are scored using a five-point Likert scale (1 = *strongly disagree* to 5 = *strongly agree*) [91]*.* In our pilot study, the Cronbach alpha for these scales were .94, .96 and .97, respectively [5,33].

##### Sociocultural relevance

Adolescents respond to statements about identification and relevance of the lesson. A sample item is “the module struck a chord with my own life.” Items are scored using a five-point Likert scale (1 = *strongly disagree* to 5 = *strongly agree*). In our pilot study, the Cronbach alpha was .96 [5,33].

##### Perceived benefits of cognitive behavioral principles

Perceived benefits of the cognitive behavioral principles taught in the intervention are measured by five questions, which are rated by adolescents on a ten-point Likert scale (1 = *very unhelpful* to 10 = *very helpful*), with higher scores indicating a stronger perceived benefit for learning cognitive behavioral therapeutic techniques [92]. A sample item is, “change my behaviors in ways that have improved my mood.” In our pilot study, Cronbach alpha was .92 [5,33].

##### Perceived benefits of interpersonal principles

Perceived benefits of the interpersonal principles taught in the intervention are measured by four questions (for example, “express my feelings and reactions to important people in my life”), to which adolescents respond on a ten-point Likert scale (1 = *very unhelpful* to 10 = *very helpful*), with higher scores indicating a stronger perceived benefit for learning interpersonal therapeutic techniques [92]. In our pilot study, the Cronbach alpha was .85 [5,33].

##### General information sheet

Participants provide demographic information about themselves at baseline and follow-up assessment points. Information collected includes age, date of birth, sex, height, weight, address, phone number(s), email address(s), race/ethnicity, school grades completed, parental marital status, family composition, and number of lifetime moves derived from the Four-Factor Hollingshead measure of social status.

##### Teen Behavior Questionnaire

The Teen Behavior Questionnaire (TBS) is a self-report questionnaire regarding diet, exercise, religion, and internet use. Questions vary in format, such as open ended, yes/no, and contain Likert scales of frequency (for example, “never” to “multiple times per day”).

##### Social Adjustment Scale

The Social Adjustment Scale (SAS-SR) is a self-report measure containing 36 items. The measure is designed to evaluate behaviors at school, with peers, and at home over the past 2 weeks (for example, “How many days of classes did you miss in the last 2 weeks?”) using a five-point ordinal scale (for example, “No days missed” to “I did not go to classes at all”).

##### Beck Hopelessness Scale

The Beck Hopelessness Scale (BHS) is a 20-item, true-false measure of the extent to which individuals are pessimistic about their future. The BHS has been shown to predict dropout from psychosocial treatment and poorer treatment response. The BHS has strong psychometric properties in adolescent samples; Cronbach’s alpha is .93 [93].

#### Safety

##### Suicide ideation scale

This is a scale used to assess the adolescent’s current level of suicidal tendencies. This measure is only used in response to adolescent reports of suicidal thinking. An example of a question asked is: If you were to weigh your reasons for living against dying, how would they compare? 0: Living outweighing dying; 1: about equal; and 2: for dying outweighing living [94].

##### Suicide intent scale

This measure is used when an adolescent has made a suicide attempt. It yields information about the seriousness of the attempt. An example of as question asked is: Did you make it hard for people to find you and to help you? 0: No precautions; 1: Passive precautions (for example, avoiding others but doing nothing to prevent their intervention); and 2: Active precautions, such as locking doors [95].

##### Lethality scale

This is a measure of the lethality of past suicide attempts, as reported by the adolescent. This does not have questions, but rather examples on a numerical scale based on intent scale. For example: 2.0: Death is improbable as an outcome of the act; if it occurs it is probably due to unforeseen secondary effects. Frequently the act is done in a public setting or is reported by the person or by others. While medical aid may be warranted, it is not required for survival. Or 10.0: Death is almost a certainty, regardless of the circumstances or interventions by an outside agent [96].

#### Adherence and log files

##### Time

Our time measures will be total time on the website in minutes, total time spent on story components in minutes, and duration of website use in days. To calculate the total time on the website in minutes, we will collect the time stamps of the first and last page loaded for each session (that is, each time a participant loads a module) and subtract the starting from the final time stamp. To avoid measuring time spent on other open pages, the length of time for each session will be capped at 7 minutes, the estimated amount of time required to review the material with a fifth grade reading level. The lengths of all sessions will be combined to create a variable for total time spent on the website measured in minutes. To calculate the time spent on the story component of the intervention in minutes, we will use a similar algorithm that subtracts the timestamp from when the story component is opened from the timestamp of when the following page is loaded. To calculate the duration of website use (the total number of days a participant spends on the intervention), we will count the number of days from the first login until the last day of the participant’s activity on the website.

##### Modules

For every participant, we will record the total number of modules completed. A module is considered complete if at least one exercise in that module is completed and/or the short online survey offered at the end of the module is completed.

##### Sessions

We define a session as the discrete occasion when a participant logs onto the website. Total number of sessions for each participant will be manually calculated by adding the total number of sessions per participant (if there was no activity after logging in, a participant is logged out after 7 minutes, but it still will be counted as a session).

##### Exercises

Exercise measures (percentage of exercises completed and number of characters typed) are used to measure the active behavior of participants. For each participant, we will calculate the percentage of exercises completed by adding the number of exercises completed across all modules and dividing this by the total number of available exercises. To measure the degree to which each exercise is completed, we will count the total number of characters typed by each participant across all modules.

### Statistical analysis

#### Survival analyses

Preceding the main analysis of whether the rate of MDD onset varies by intervention condition, we will test for constant hazard over time. If this test is not significant at 0.10 will use Cox proportional hazards, adjusted for age and sex, to compute the hazard ratio for adolescent major depressive episodes for the intervention group in comparison to the HE group. We will also test whether time to MDD depends on the interaction of intervention condition by baseline level of symptoms. If the test or diagnostics reveal nonproportional hazards (that is, there is evidence of differential effect over time), we will include this nonproportionality in analyses. We have used such limited “model fitting” tests without regard to the magnitude of intervention effect in previous analyses that still preserve the Type I error rate [97], and will follow multiple comparison procedures we have used previously to identify time intervals where significant effects are found [88].

#### Longitudinal data analysis

We test the hypothesis that the intervention affects the course of depressive symptoms and other measures across time. Our general method will be to use growth curve modeling with continuous outcomes to examine how the intervention changes adolescent depressive symptoms, vulnerability factors, educational impairment, and quality of life. Simple comparisons will be calculated as appropriate (for example, number of depressed days [98]), and survival curve modeling will be used for binary outcomes for prevalence of disorders [44,99-103]. Guided by our methods development on growth models, we will address missing data using full information maximum likelihood and multiple imputation, which provide improvement over “last observation carried forward” and other less refined methods [104-106]. We will also examine whether or not there are variations in growth trajectories by introducing covariate interactions and mixtures to growth models, techniques that we and our colleagues have developed [97,107-110]. These more complex models can characterize quantitatively differential response, as well as qualitative changes, such as recurrence [111]. All such models will be preceded by careful model checking [67,93,107,112].

#### Mediator analysis

In addition to examination of whether the intervention impact varies as a function of baseline symptoms, we will evaluate potential mediators including change in adolescent motivation, principles (intervention experience characteristics) of effective preventive interventions (adherence/dose, positive relationships, training and socio-cultural relevance, as reported by the adolescent) and changes in adolescent vulnerability and protective factors (social support, automatic negative thoughts, depressed mood). Based on our recent work on a comparison of analytic models for mediation with nonlinear models [113], we will use the “product of coefficients” method to conduct tests of mediation and the bootstrap method for accurate confidence intervals [114]. To examine the effects of adolescent participation with the website, we will conduct complier average causal effect (CACE) analyses that include covariates predicting participation status [115-118].

#### Moderator analysis

We expect to conduct tests of moderation in eight domains: (a) adolescent demographic and cultural factors [119-121], (b) adolescent vulnerability factors and adverse events [51], (c) adolescent motivation and attitudes [62], (d) adolescent relationship with the provider, (e) parent and child comorbid psychopathology, (f) adolescent treatment before and during the study [122,123], (g) level of adolescent adherence/participation, and (h) study site. We will use subgroup analyses and tests of interaction to evaluate moderator effects. We anticipate conducting several different subgroup analyses including the following: (a) adolescent ethnicity, sex and age; (b) neighborhood socioeconomic level; (c) adolescent level of risk, including risk stratified by varying levels of vulnerability from different sources (for example, family, cognitive or social risk); and (d) practice factors (provider motivational interview quality, practice type).

#### Sample size analysis

We conducted a series of power/sample size calculations through a SAS macro [124] under realistic alternatives to address impact on depressive symptoms. We require 200 adolescent subjects per intervention condition to achieve 80% power based on a conservative application of our pilot study findings from the CATCH-IT trial [33]. These calculations assume that in the control group, 72% are free from depression after one year follow-up, and the second year continues to follow the same exponential rate for controls; for intervention, the hazard is a constant ratio of 0.62, and an attrition rate of 7% for each of the first four quarters and 2% for each of the second four quarters. Note that the estimated hazard rate of 0.62 is two-thirds of that we observed in this previous study. There are concerns raised about the use of pilot study data to assess power, even using a lower value than we observed [125], so we conducted other power calculations based on the data from a similar study [45]. In their study, they found a departure from exponential and disproportional hazards of intervention over time. Assuming the same attrition rate as we found above, a study with 400 subjects would produce 80% power when the effect of CATCH-IT was only 60% at every interval of time relative to the CBT hazard rate in the Clarke study. Thus, we expect to have sufficient power even if the overall benefit is 60% of what was obtained by Clarke.

#### Previous outcomes

Pilot studies of earlier versions of CATCH-IT demonstrated favorable changes in adolescent depressive symptoms, dysfunctional thinking, and social support postintervention. In a randomized clinical trial, where adolescents were randomized to either Internet intervention + Motivational Interview with Primary Care Provider(PCP) or Internet intervention + Brief Advice with Primary Care Provider, significant changes in adolescent depressed mood, social support, and number of depressive episodes were observed from baseline to 12-month follow-up [5,33,126,127], and fewer depressive episodes were diagnosed by PCP post-intervention in adolescents who received Motivational Interviews than in adolescents who received minimal provider support (brief advice condition).

#### Current trial status

As of September 2014, 4,405 (N = 2,369 in Chicago, N = 2,036 in Boston) adolescents have been identified through the two-question screener or recruitment letter as potentially eligible for the study (recruiting in Chicago began several months prior to recruitment in Boston). Two hundred thirty-four adolescents (N = 130 in Chicago, N = 104 in Boston) have been enrolled. The average age of adolescent participants is currently 14.87 (*SD* = 1.42 years).

## Discussion

Major depression is a common disorder that causes significant morbidity, lost productivity, and is associated with an increased risk of suicide. The first episode usually occurs in adolescence. There are currently no widely available, low cost, effective and acceptable depression prevention interventions for adolescents. The development of a feasible, cost-effective, Internet-based preventive intervention for this age group would be of great value. We describe here a protocol of a study currently in the field, designed to determine if a low-cost, universally available (Internet-based) and population-based (primary care screening and engagement), theory-grounded depression preventive intervention is superior to a health education attention control intervention in preventing onset of major depression and/or symptoms of anxiety, behavioral disorders, and substance abuse.

This trial design has several unique features that will distinguish it from many other recently completed and ongoing trials. While many school-based trials have been undertaken, there is currently no systematic approach to connect at-risk youth with self-directed depression prevention programs in a medical setting [34,128,129]. Medical settings may be particularly important, because of an increased interest within the healthcare field in containing costs through early intervention to prevent chronic disease [130]. This trial undertakes the complex public health task of identifying at-risk individuals through mass screening of the general primary care population, rather than solely relying on volunteers recruited over the Internet, as is done for many Internet trials [131,132]. The trial design includes both traditional continuous measure of mental disorder symptoms most often used in Internet studies, and structured clinical interviews of presence of depressive disorder or episode [5,39].

The evaluated intervention, CATCH-IT, has several distinguishing features. CATCH-IT was developed using a longitudinal, interactive revision model, attending to both user experience decade and single versions [40,55,131,133]. CATCH-IT was designed from the ground-up to appeal to hard-to-persuade, primary care patients who prefer “natural” and “health promotion” interventions over overtly psychological “treatment” [24,47,55,62]. Many Internet interventions either provide no supportive structure or rely primarily on study staff to perform coaching functions; CATCH-IT seeks to systematically integrate the adolescent engagement and motivation process into the existent primary care organization [38,55,134]. Like many successful intervention models, it incorporates both carefully structured direct human contact, or “coaching,” and user-friendly interface intended to maximize adolescents’ meaningful engagement with the intervention [40,135].

Fielding multicomponent randomized clinical trials for the prevention of mental disorders in the community setting has high public health relevance, but important limitations must be considered with regard to study design. Recruited participants via a primary care screening process may include adolescent participants with limited motivation to engage in the intervention, compared to participants in other intervention studies where adolescents self-select to participate [132,136]. Lower motivation of study participants may negatively impact intervention outcomes, perhaps directly through low motivation by decreasing website use, or indirectly, negatively impacting outcomes due to differing levels of resiliency or beliefs about mental health [138]. Similarly, placebos of any kind may produce effects of relatively similar size to active psychotherapy interventions, incurring the risk of failure to demonstrate significant differences between groups [137].

Additional limitations may be related to pediatric practices, the control condition model, and implementation. There may be meaningful variations between practices and sites as to levels of intervention fidelity (for example, motivational interview quality) and adherence/dose (Internet site) that could impact study outcomes [138]. Additionally, the HE control condition, while derived from a non-psychoactive model in adults, may perform differently in adolescents and children. Finally, there is a need to study the entire implementation process, all of which can impact outcomes [138]. For example, implementation at a practice can vary greatly depending on whether or not the practice has competing demands for attention (for example, a simultaneous implementation of health reform measures, an additional research study, or a new electronic health records system) during the implementation or active portion of the study.

This study provides a model for what may be a new generation of interventions that blend substantial computer-based instruction with limited human contact to intervene to prevent mental disorders, or what can be called a behavioral vaccine [124,139]. Because of the potential for broad generalizability of this model, from an intervention efficacy standpoint, the results of this study are important because they will help to develop the guidelines for preventive interventions with youth at-risk for the development of depressive and other mental disorders. The study could lay the foundation in terms of implementation “scaffolding” for an entire model of primary care, technology-based prevention of common behavioral disorders - reshaping health systems to embrace cost-saving, disease preventing, and life experience enhancing technologies. As the study progresses, the investigators will need to carefully weigh trade-offs between these highly valued outcomes to optimize the performance of the study.

## Trial status

This manuscript reports the protocol for an ongoing clinical trial, for which patient recruitment is currently ongoing.

## Abbreviations

BA: behavioral activation
CATCH-IT: Competent Adults Transition with Cognitive Behavioral Humanistic and Interpersonal Training
CBT: Cognitive Behavioral Therapy
CES-D: Center for Epidemiologic Studies Depression
HE: Health Education
IPT: Interpersonal Psychotherapy
K-SADS: Kiddie Schedule of Affective Disorders
MDD: Major Depressive Disorder
PCP: Primary Care Provider
SAS-SR: Social Adjustment Scale - self report
BHS: Beck Hopelessness Scale
